# Association between hypertension and risk of respiratory failure and mortality in patients with COVID-19: a retrospective cohort study with predictive model development

**DOI:** 10.3389/fmed.2026.1825373

**Published:** 2026-05-07

**Authors:** Xiaoqing Hu, Kailong Ye, Bifang Miao, Yiqun Dai, Lianghua Guo, Linmiao Zeng, Xiu Chen, Chuanqi Zhu, Qinghua Zhang, Bin Song, Jianhong Xiao

**Affiliations:** 1 Department of Respiratory Medicine, Mindong Hospital Affiliated to Fujian Medical University, Ningde, China; 2 Department of Obstetrics, Mindong Hospital Affiliated to Fujian Medical University, Ningde, China

**Keywords:** COVID-19, hypertension, in-hospital mortality, LASSO regression, predictive model, respiratory failure, SARS-CoV-2

## Abstract

**Background:**

Hypertension is highly prevalent among patients with Coronavirus Disease 2019 (COVID-19), yet its impact on respiratory failure and mortality remains controversial. Our objective was to evaluate the association between hypertension and adverse outcomes in COVID-19 patients and to develop predictive models for risk stratification.

**Objective:**

To investigate the association between hypertension and respiratory failure or mortality in patients with COVID-19: mediation analysis of laboratory biomarkers and development of a risk prediction model for adverse outcomes in hypertensive individuals with COVID-19.

**Methods:**

We retrospectively analyzed clinical data from hospitalized COVID-19 patients. After 1:1 propensity score matching (PSM) of hypertensive and non-hypertensive patients, multivariable logistic regression assessed hypertension’s independent association with respiratory failure and in-hospital mortality. Mediation and subgroup analyses explored biomarker mediators and effect modifiers. Least Absolute Shrinkage and Selection Operator (A LASSO)—selected risk prediction model was validated by Area Under the ROC Curve (ROC-AUC) analysis.

**Results:**

Multivariable logistic regression showed that hypertension was independently associated with increased risks of respiratory failure (OR = 2.196, 95% CI: 1.170–4.120; *P* = 0.014) and in-hospital mortality (OR = 2.945, 95% CI: 1.420–6.107; *P* = 0.004). Mediation analysis revealed that eosinophil count and Estimated Glomerular Filtration Rate (eGFR) partially mediated these associations: for respiratory failure, they accounted for 44.5 and 15.6% of the total effect, respectively; for mortality, 50.0 and 16.7%. LASSO-based prediction models demonstrated an AUC of 0.788 for respiratory failure (using neutrophil count, C-Reactive Protein (CRP), Blood Urea Nitrogen (BUN), and albumin) and 0.694 for mortality (using neutrophil count and BUN).

**Conclusion:**

Compared with non-hypertensive COVID-19 patients, those with hypertension had significantly higher risks of respiratory failure and in-hospital mortality. Eosinophil count and eGFR significantly mediated these associations. A multi-marker model—combining neutrophil count, CRP, BUN, and albumin—showed good predictive performance for respiratory failure, supporting early risk identification and precision clinical decision-making.

## 1 Introduction

Coronavirus disease 2019 (COVID-19) is an acute respiratory infectious disease caused by severe acute respiratory syndrome coronavirus 2 (SARS-CoV-2). Its primary modes of transmission include respiratory droplets, close contact, and aerosol transmission in relatively enclosed environments (1, 2). Typical clinical manifestations mainly include cough, fever, dry throat, sore throat, fatigue, and myalgia; severe cases often develop dyspnea and/or hypoxemia within 1 week after disease onset (1–3). Age ≥ 65 years, comorbidities such as hypertension, diabetes, and kidney disease, as well as immunosuppression, are all recognized high-risk factors for disease progression to severe COVID-19 (1, 2, 4). According to the World Health Organization (WHO), as of 28 September 2025, a cumulative total of 779 million confirmed COVID-19 cases have been reported globally (5). Specifically, China has documented a cumulative incidence of 99.4 million cases, placing it second globally in terms of total case burden (5). Given the current viral evolution trends and disease burden, the WHO continues to classify COVID-19 as posing a “high global public health risk” (6), underscoring the ongoing need for in-depth research and practical efforts to continuously refine prevention, control, and clinical management strategies in response to this enduring public health challenge (7).

Hypertension, the most prevalent cardiovascular disease worldwide, affects approximately 1.4 billion individuals globally, yet only 14% of patients achieve adequate blood pressure control (8, 9). In the context of COVID-19, hypertension is consistently identified as one of the most frequent comorbidities and is disproportionately observed among patients with severe disease (10–12). Numerous studies have demonstrated that COVID-19 patients with comorbid hypertension face a significantly elevated risk of disease progression and mortality compared to their non-hypertensive counterparts (4, 13–16). Nevertheless, uncertainty persists as to whether hypertension constitutes an independent risk factor after rigorous adjustment for potential confounders, and the underlying biological mechanisms and mediating pathways remain incompletely characterized (17, 18).

Emerging evidence indicates that specific laboratory parameters, particularly reduced eosinophil counts and decreased estimated glomerular filtration rate (eGFR), are closely associated with disease severity and adverse prognosis in COVID-19 (19–22). Eosinopenia may reflect underlying immune dysregulation, whereas a decline in eGFR may signal renal impairment; both are thought to contribute to the pathophysiology of severe COVID-19. However, some investigations suggest that the apparent effect of hypertension on COVID-19 outcomes may be attenuated following rigorous adjustment for confounders such as age (17, 18). Thus, multifactorial-adjusted mediation analysis is warranted to delineate the precise pathways linking hypertension to prognosis and to facilitate more accurate risk stratification and personalized intervention.

Based on these considerations, our retrospective cohort study employed propensity score matching (PSM) to minimize selection bias and enhance comparability between hypertensive and non-hypertensive groups. After adjusting for key confounders, we sought to: (1) evaluate the independent association between hypertension and the risks of respiratory failure and in-hospital mortality; (2) explore potential mediating pathways through formal mediation analysis; and (3) assess effect modification via subgroup analyses. Finally, a risk prediction model was constructed using Least Absolute Shrinkage and Selection Operator (LASSO) regression to facilitate the early identification of high-risk individuals and support precision clinical decision-making, while mitigating overfitting.

## 2 Methods

### 2.1 Study design and participants

This study aimed to investigate the association between hypertension and the risk of respiratory failure and mortality in patients with COVID-19, to examine the mediating role of laboratory biomarkers, and to develop a risk prediction model for adverse outcomes specifically in patients with comorbid hypertension. This research utilized a monocentric retrospective cohort methodology. COVID-19 patients hospitalized in the Department of Respiratory and Critical Care Medicine at Mindong Hospital in Ningde, Fujian Province, from October 2022 to May 2023 were included. Ethical approval was granted to this study by the hospital’s Ethics Committee.

*Inclusion criteria*: (1) Inclusion required a confirmed diagnosis of COVID-19 in accordance with the WHO clinical management guidelines (1); (2) Age > 18 years; (3) First-time SARS-CoV-2 infection; (4) Complete clinical data available. *Exclusion criteria*: (1) Pregnant or lactating women; (2) Prior history of COVID-19 infection; (3) Severe acute cardiovascular or cerebrovascular disease; (4) Severe immunodeficiency diseases; (5) Chronic kidney disease [baseline estimated glomerular filtration rat (eGFR) < 60 mL/min/1.73 m^2^]; (6) Liver cirrhosis; (7) Missing key clinical data.

### 2.2 Data collection

Data on patients’ demographic characteristics, medical history, initial laboratory results during hospitalization, and clinical outcomes were systematically collected. Baseline characteristics included gender, age, body mass index (BMI), smoking status (defined as current or former smoking), alcohol consumption, hypertension, chronic obstructive pulmonary disease (COPD), and diabetes mellitus. Laboratory parameters included the following 47 indicators:Complete blood count (CBC): white blood cells (WBC), neutrophils (NEUT), lymphocytes, neutrophil-to-lymphocyte ratio (NLR), monocytes (MO), eosinophils (Eos), basophil count (BA), red blood cells (RBC), hemoglobin (Hb), mean corpuscular volume (MCV), mean corpuscular hemoglobin (MCH), mean corpuscular hemoglobin concentration (MCHC), hematocrit (Hct), and platelets (PLT), Inflammatory markers: C-reactive protein (CRP) and procalcitonin (PCT), Comprehensive metabolic panel: alanine aminotransferase (ALT), aspartate aminotransferase (AST), albumin (ABL), globulin (GLB), total bilirubin (TBIL), direct bilirubin (DBIL), indirect bilirubin (IBIL), lactate dehydrogenase (LDH), creatine kinase (CK), Creatine Kinase-Myocardial Band (CK-MB), creatinine (CREA), estimated glomerular filtration rate (eGFR), blood urea nitrogen (BUN), glucose (GLU), triglycerides (TG), total cholesterol (CHO), potassium (K^+^), sodium (Na^+^), chloride (Cl^–^), calcium (Ca^2+^), phosphate (P), anion gap (AG), and osmotic pressure (OSMO); Coagulation profile: prothrombin time (PT), thrombin time (TT), activated partial thromboplastin time (APTT), international normalized ratio (INR), and fibrinogen (FIB); Additional biomarkers: D-dimer, troponin, and B-type natriuretic peptide (BNP). The primary outcomes were respiratory failure and in-hospital all-cause mortality. Respiratory failure was defined as a partial pressure of oxygen (PaO2) < 60 mmHg or the requirement for non-invasive or invasive mechanical ventilation.

### 2.3 Statistical analysis

To minimize selection bias and confounding, propensity score matching (PSM) was performed using sex and age as matching variables. Nearest-neighbor matching with a caliper width of 0.15 was applied at a 1:1 ratio. Matching balance was assessed using standardized mean differences (SMDs), with SMD < 0.1 indicating adequate covariate balance. Descriptive analyses were conducted on the PSM-matched cohort. Continuous variables were expressed as mean ± SD (if normally distributed) or median (IQR) (otherwise); categorical variables as frequencies (%). Group comparisons used independent-samples *t*-tests, Mann–Whitney U tests, or chi-square/Fisher’s exact tests, as appropriate. Descriptive analysis was performed on the cohort after propensity score matching. The normality of continuous variables was assessed using the Kolmogorov–Smirnov test. For normally distributed data with homogeneity of variance, independent samples t-tests were used for comparison, and results are presented as mean ± standard deviation; otherwise, the Mann–Whitney U test was employed, and results are expressed as the interquartile range. Categorical variables are presented as frequencies (percentages), and inter-group comparisons were conducted using the chi-square test or Fisher’s exact test. Binary multivariable logistic regression was used to evaluate the association of hypertension with respiratory failure and in-hospital mortality in COVID-19 patients, adjusting for potential confounders in a stepwise manner. Subgroup analyses were conducted to assess potential effect modification by age, sex, and smoking status on the association between hypertension and risks of respiratory failure and in-hospital mortality in COVID-19 patients, thereby identifying potential effect modifiers. The mediating roles of laboratory biomarkers in the associations between hypertension and risks of respiratory failure and in-hospital mortality in COVID-19 patients were evaluated using the bootstrap method (1,000 replications), with estimates of direct effects, indirect effects, and proportion mediated reported. The intermediary contributions of eosinophil count and eGFR to the hypertension–respiratory failure association in COVID-19 patients were examined via mediation analysis. This procedure relied on a sequential regression framework comprising three components. First, the total effect of hypertension on respiratory failure was estimated. Second, the independent associations of hypertension with each mediator were evaluated. Third, hypertension and the mediators were combined within a single model to partition the total effect into direct and indirect components. Inference for the indirect pathways was based on a bootstrap resampling technique with 1,000 iterations. Mediation was considered statistically supported when the 95% confidence intervals excluded zero. Variable selection was performed using least absolute shrinkage and selection operator (LASSO) regression. The optimal regularization parameter (λ) was determined via 10-fold cross-validation. The model corresponding to λ1se was selected to enhance generalizability. Logistic regression models incorporating the LASSO-selected predictors were then constructed to predict the risk of respiratory failure and in-hospital mortality in COVID-19 patients with hypertension. Model performance was assessed using the area under the receiver operating characteristic curve (AUC). All statistical analyses were performed using SPSS version 26.0 and R software (version 4.5.0; R Foundation for Statistical Computing, Vienna, Austria). Two-sided tests were used, and a *P* < 0.05 was considered statistically significant.

**FIGURE 1:**
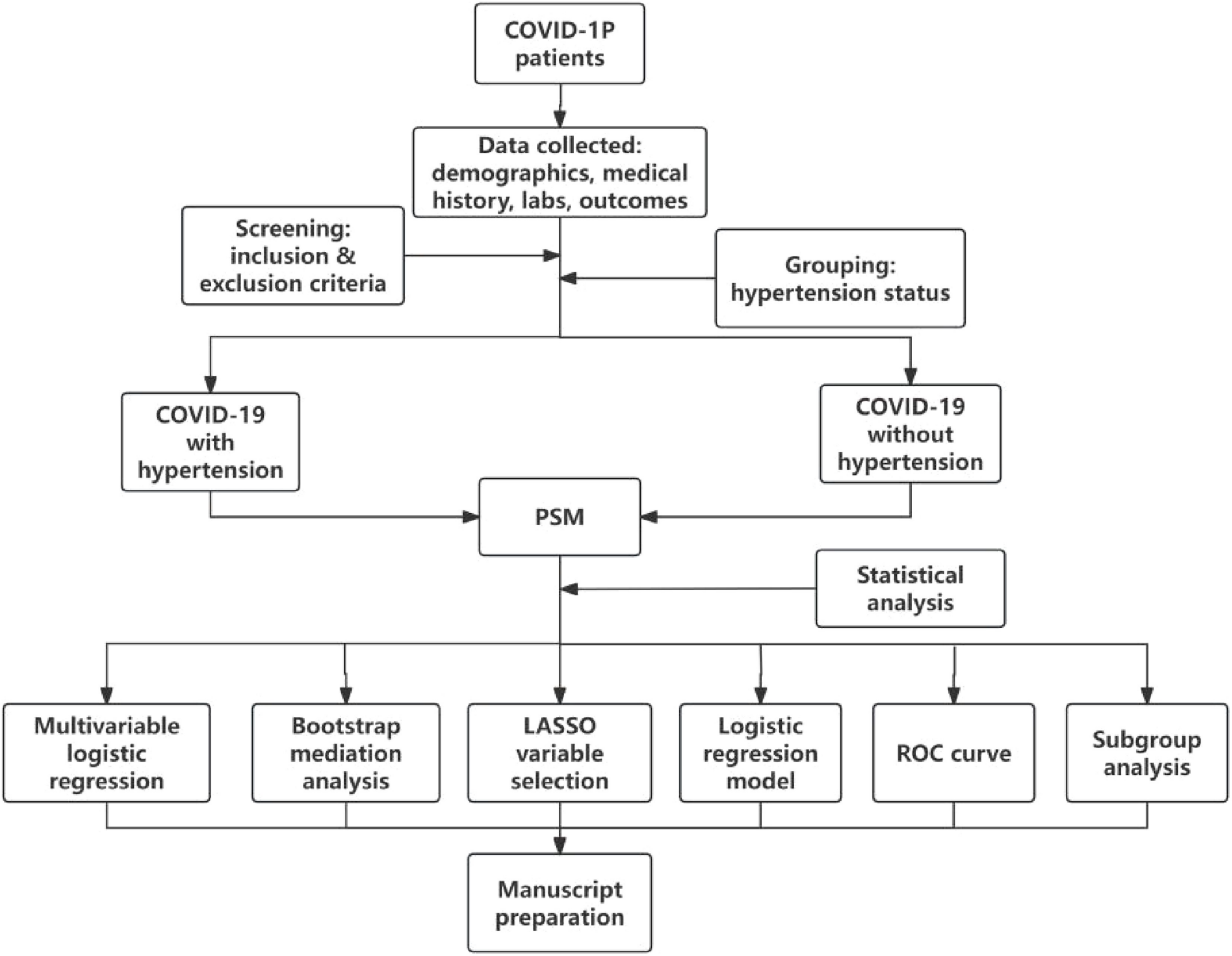
Technical flowchart.

Flowchart of participant selection in this study (Figure 1).

## 3 Results

### 3.1 Propensity score matching (PSM)

#### 3.1.1 PSM balance diagnostics

**FIGURE 2:**
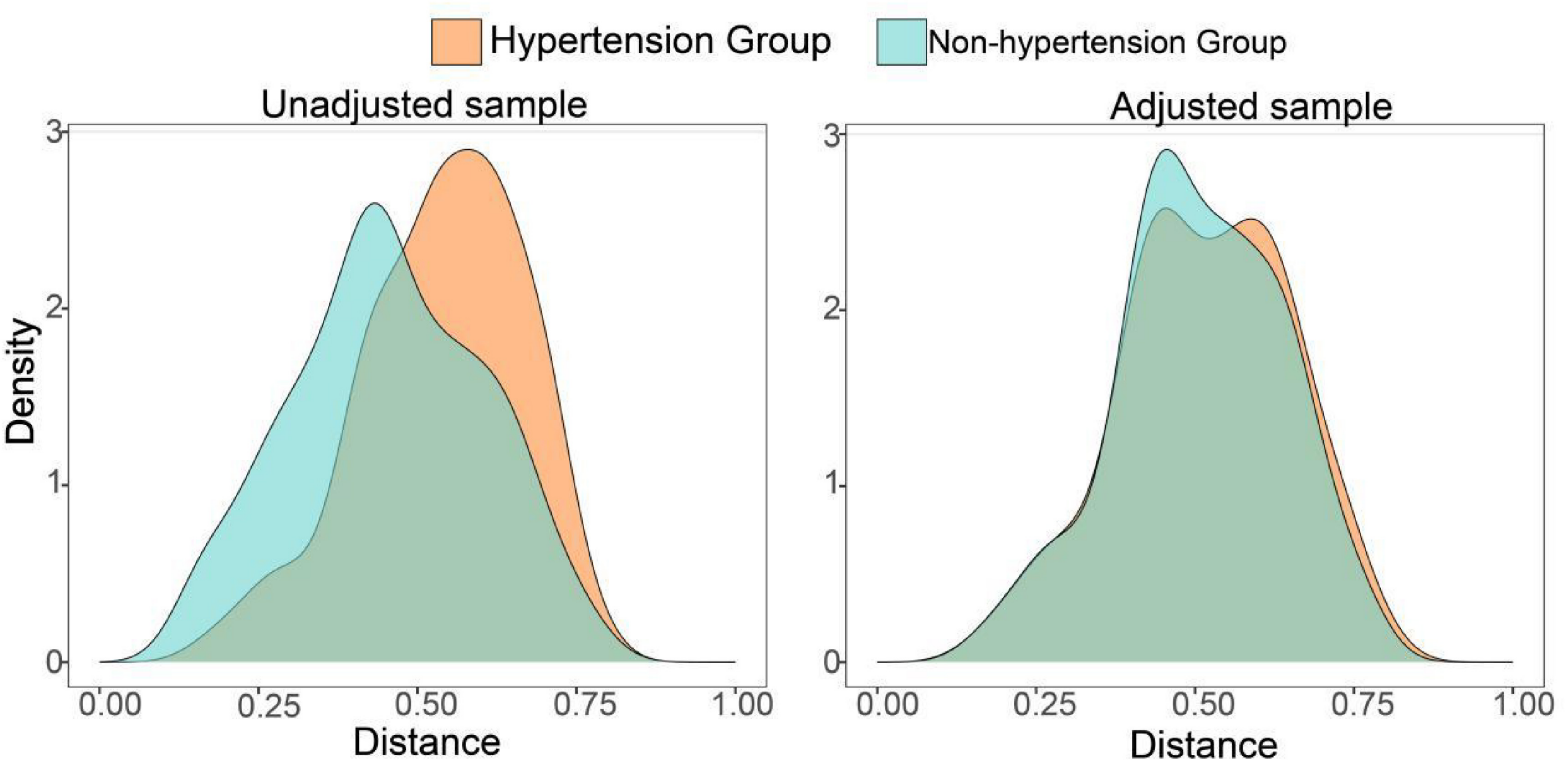
Distribution of balance before and after PSM matching.

**FIGURE 3:**
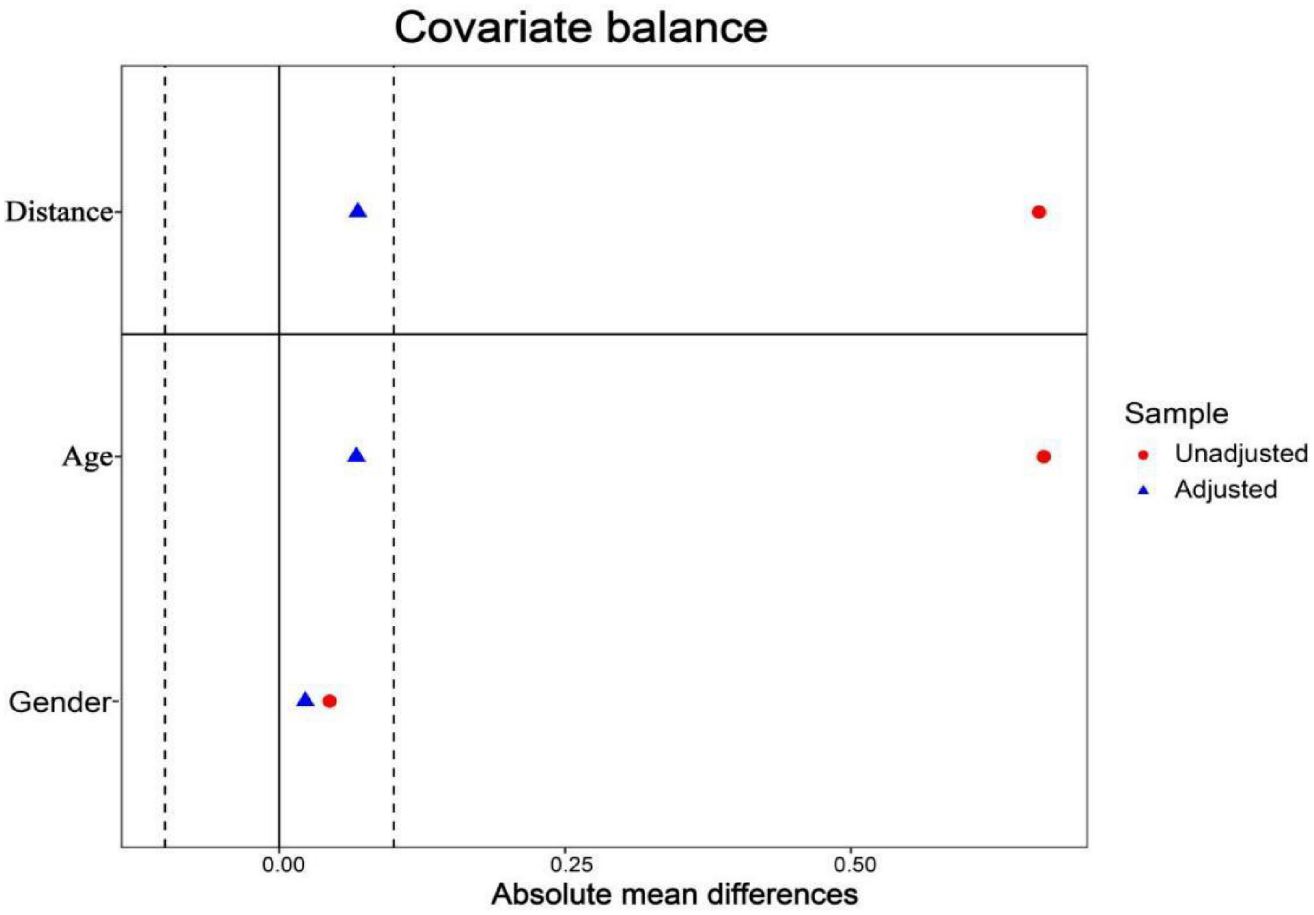
Distribution of absolute mean difference before and after PSM matching. “Unadjusted” refers to the unmatched sample. “Adjusted” refers to the matched sample.

A total of 357 eligible COVID-19 patients were enrolled; after PSM, 132 patients were included in each group: COVID-19 with hypertension and COVID-19 without hypertension. After matching, the absolute values of SMD for all covariates were < 0.1, indicating good balance between groups and an ideal matching effect (Figure 2 and Table 1). Furthermore, the substantial overlap in propensity score distributions between the two groups after matching (Figure 3) confirms the comparability of the matched cohorts and supports the reliability of the statistical balance assessment (SMD < 0.1).

**TABLE 1:** PSM balance diagnosis.

Variable	Variable type	Pre-matching difference (SMD)	Post-matching difference (SMD)	Post-matching Balance (SMD < 0.1)
Distance	Propensity score	0.6645	0.0687	✓ (< 0.1)
Age	Continuous variable	0.6075	0.0651	✓ (< 0.1)
Gender	Binary variable	0.0904	0.0469	✓ (< 0.1)

“Pre-matching difference (SMD)” refers to the standardized mean difference before propensity score matching. “Post-matching difference (SMD)” refers to the standardized mean difference after propensity score matching.

#### 3.1.2 Baseline characteristics of the study population

Baseline characteristics of the study population after PSM are presented in Table 2. Results showed that after matching, the hypertension group had significantly higher rates of diabetes (23.5% vs. 12.9%, *P* = 0.025), overweight/obesity (38.6% vs. 17.4%, *P* < 0.001), respiratory failure (28.8% vs. 15.9%, *P* = 0.012), and mortality (23.5% vs. 10.6%, *P* = 0.005) compared with the non-hypertension group. Baseline characteristics, including age, sex, smoking status, alcohol use, and COPD history, were comparable between the two cohorts (Table 2).

**TABLE 2:** Baseline characteristics after propensity score matching.

Variable	Hypertension Group (*N* = 132)	Non-hypertension Group (*N* = 132)	χ ^2^/t/z	*P*
Age (years)			0.029	0.865^a^
<65	21(15.9)	20(15.2)		
≥65	111(84.1)	112(84.8)		
Gender			0.145	0.703^a^
Female	48(36.4)	51(38.6)		
Male	84(63.6)	81(61.4)		
Smoke			0.063	0.802^a^
No	52(39.4)	54(40.9)		
Yes	80(60.6)	78(59.1)		
Alcohol			2.729	0.213^b^
No	131(99.2)	127(96.2)		
Yes	1(0.8)	5(3.8)		
Respiratory failure			6.308	0.012^a^
No	94(71.2)	111(84.1)		
Yes	38(28.8)	21(15.9)		
Death			7.742	0.005^a^
No	101(76.5)	118(89.4)		
Yes	31(23.5)	14(10.6)		
Diabetes			4.991	0.025^a^
No	101(76.5)	115(87.1)		
Yes	31(23.5)	17(12.9)		
COPD			0.007	0.792^a^
No	89(67.4)	91(68.9)		
Yes	43(32.6)	41(31.1)		
BMI (kg/m^2^)			14.721	<0.001^a^
Normal	81(61.4)	109(82.6)		
Overweight or obesity	51(38.6)	23(17.4)		
FIB (g/L)	4.3 ± 1.1	4.4 ± 1.0	0.638	0.524^c^
CL^–^ (mmol/L)	100.7 ± 5.4	99.4 ± 5.1	–1.930	0.055^c^
Ca^2+^ (mmol/L)	2.1 ± 0.1	2.1 ± 0.1	0.636	0.525^c^
P (mmol/L)	0.9 ± 0.2	1.0 ± 0.3	0.447	0.656^c^
eGFR (mL/min/1.73 m^2^)	78.1 ± 22.6	86.0 ± 22.7	2.563	0.011^c^
MCV (fL)	90.9(87.1, 93.8)	88.8(86.2, 91.5)	–1.911	0.056^d^
MCH (pg)	29.8(28.8, 31.4)	29.3(28.6, 30.5)	–2.567	0.010^d^
MCHC (g/L)	332.0(320.0, 338.0)	326.0(322.0, 336.0)	–2.675	0.007^d^
PLT (109/L)	176.0(144.0, 269.0)	193.0(144.0, 253.0)	–0.636	0.525^d^
CREA (μmol/L)	70.0(60.0, 89.0)	66.3(52.0, 77.7)	–0.850	0.395^d^
GLU (mmol/L)	6.2(5.0, 7.9)	5.1(4.7, 7.1)	–2.647	0.008^d^
LDH (U/L)	213.0(186.0, 294.0)	190.0(162.0, 277.0)	–3.495	0.000^d^
CK (U/L)	70.0(35.0, 119.0)	71.0(41.0, 114.0)	–1.297	0.195^d^
CKMB (U/L)	15.0(11.0, 19.0)	12.4(8.0, 17.0)	–0.542	0.588^d^
TG (mmol/L)	1.2(1.0, 1.6)	1.0(0.9, 1.2)	–0.709	0.087^d^
CHO (mmol/L)	4.0(3.6, 4.7)	3.9(3.7, 4.3)	–0.447	0.655^d^
PT (s)	11.8(11.3, 12.4)	11.9(11.5, 12.5)	–0.151	0.880^d^
INR	1.1(1.0, 1.1)	1.1(1.0, 1.1)	–0.077	0.939^d^
APTT (s)	30.5(28.8, 33.8)	31.2(28.8, 33.7)	–2.227	0.026^d^
TT (s)	13.4(13.2, 14.3)	13.1(12.3, 13.4)	–2.453	0.014^d^
WBC (109/L)	5.6(4.9, 7.4)	6.3(5.0, 10.4)	–2.440	0.015^d^
NEUT (109/L)	4.0(3.3, 5.5)	3.9(3.3, 8.1)	–1.477	0.140^d^
Lymphocytes (109/L)	1.1(0.6, 1.3)	1.0(0.7, 1.5)	–0.542	0.588^d^
NLR	3.9(3.1, 7.6)	4.7(2.6, 10.6)	–0.605	0.545^d^
MO (109/L)	0.6(0.4, 0.8)	0.5(0.4, 0.9)	–0.873	0.382^d^
EO (109/L)	0.01(0.00, 0.03)	0.01(0.00, 0.06)	–0.712	0.477^d^
BA (109/L)	0.01(0.01, 0.02)	0.01(0.01, 0.02)	–3.853	0.000^d^
RBC (1012/L)	4.3(3.7, 4.7)	4.5(4.0, 4.9)	–0.628	0.530^d^
HB (g/L)	131.0(113.0, 137.0)	132.0(122.0, 140.0)	–0.681	0.496^d^
CRP (mg/L)	33.2(14.2, 55.5)	34.2(12.3, 56.7)	–1.090	0.276^d^
ALB (g/L)	34.9(32.8, 39.9)	40.2(33.3, 41.3)	–1.715	0.086^d^
GLB (g/L)	26.3(22.6, 29.4)	27.4(24.0, 31.7)	–1.286	0.199^d^
TBIL (μmol/L)	9.1(6.8, 11.5)	10.4(8.4, 13.5)	–0.556	0.578^d^
DBIL (μmol/L)	3.3(2.3, 4.2)	4.1(2.6, 5.2)	–2.512	0.012^d^
IBIL (μmol/L)	5.9(4.6, 7.4)	5.9(5.0, 8.4)	–0.512	0.609^d^
K^+^ (mmol/L)	4.1(3.7, 4.4)	4.0(3.8, 4.4)	–1.751	0.080^d^
Na^+^ (mmol/L)	138(135.0, 141.0)	138.0(136.0, 139.0)	–0.648	0.517^d^
D-dimer (mg/L FEU)	0.7(0.5, 1.2)	0.6(0.5, 0.8)	–1.199	0.231^d^
Troponin (ng/L)	0.013(0.008, 0.023)	0.008(0.004, 0.015)	–1.892	0.058^d^
BNP (pg/mL)	96.2(57.5, 181.8)	86.5(54.7, 125.0)	–0.740	0.459^d^
PCT (ng/mL)	0.04(0.03, 0.12)	0.04(0.01, 0.05)	–1.023	0.306^d^
ALT (U/L)	25.0(14.0, 34.0)	19.0(11.0, 28.8)	–2.716	0.007^d^
AST (U/L)	27.0(20.0, 35.0)	24.0(18.0, 33.0)	–2.214	0.027^d^
HCT (%)	40.1(33.8, 41.3)	40.1(36.7, 42.5)	–0.019	0.985^d^
AG (mmol/L)	10.1(9.1, 13.0)	10.4(9.3, 13.0)	–0.097	0.923^d^
BUN (mmol/L)	4,9(4.0, 7.3)	3.9(3.5, 5.4)	–0.736	0.462^d^
OSMO (mOsm/kg)	278.0(270.0, 284.0)	277.0(269.0, 279.0)	-0.401	0.688^d^

^a^*P*-value was calculated using the chi-square test by comparing the composition ratios of categorical variables between the non-hypertensive group and the hypertensive group.

^b^*P*-value was obtained using Fisher’s exact test to compare the differences in categorical variables between the non-hypertensive group and the hypertensive group.

^c^*P*-value was calculated using the independent samples *t*-test to compare continuous variables between the non-hypertension group and the hypertension group.

^d^*P*-value was calculated using the Mann-Whitney U test to compare continuous variables between the non-hypertension group and the hypertension group.

### 3.2 Association between hypertension and risks of respiratory failure and mortality in COVID-19 patients

#### 3.2.1 Association between hypertension and respiratory failure in COVID-19

Multivariable logistic regression analysis showed that, after adjusting for potential confounders (smoking history, alcohol consumption, BMI, and diabetes), the model demonstrated good overall fit (Hosmer–Lemeshow test: χ^2^ = 6.553, *P* = 0.477). Hypertension was significantly associated with a higher risk of respiratory failure in COVID-19 patients (adjusted OR = 2.196, 95% CI: 1.17–4.12; *P* = 0.014), corresponding to a 120% increased risk (Table 3).

**TABLE 3:** Association between hypertension and risk of respiratory failure in Patients with COVID-19.

Variable		Analysis type	OR/aOR/bOR, 95%CI	*P*-value
Hypertensionl	No	Univariate analysis	1.0(reference)	–
	Yes		2.137(1.173, 3.892)	0.013
	No	Multivariate analysis (Model 1)^a^	1.0(reference)	–
	Yes		2.230(1.191, 4.175)	0.012
	No	Multivariate analysis (Model 2)^b^	1.0(reference)	–
	Yes		2.196(1.170, 4.120)	0.014
Smoking	No	Multivariate analysis (Model 1)^a^	1.0(reference)	–
	Yes		2.126(1.115, 4.051)	0.022
	No	Multivariate analysis (Model 2)^b^	1.0(reference)	–
	Yes		2.161(1.130, 4.132)	0.020
Alcohol	No	Multivariate analysis (Model 1)^a^	1.0(reference)	–
	Yes		0.998(0.111, 9.001)	0.999
	No	Multivariate analysis (Model 2)^b^	1.0(reference)	–
	Yes		0.951(0.105, 8.640)	0.964
BMI	Normal	Multivariate analysis (Model 1)^a^	1.0(reference)	–
	Overweight/Obese		0.837(0.427, 1.641)	0.604
	Normal	Multivariate analysis (Model 2)^b^	1.0(reference)	–
	Overweight/Obese		0.805(0.407, 1.592)	0.532
Diabetes	No	Multivariate analysis (Model 2)^b^	1.0(reference)	–
	Yes		1.328(0.631, 2.795)	0.455

(Model 1)^a^ refers to multivariate logistic regression analysis adjusted for “hypertension, age, and sex”.

(Model 2)^b^ refers to multivariate logistic regression analysis adjusted for “hypertension, age, sex, smoking, and alcohol consumption”.

#### 3.2.2 Association between hypertension and mortality risk in COVID-19 patients

Multivariable logistic regression analysis showed that, after adjusting for smoking history, alcohol consumption, BMI, and diabetes, the model exhibited good overall fit (Hosmer–Lemeshow test: χ^2^ = 1.828, *P* = 0.969). Hypertension was significantly associated with increased mortality risk in COVID-19 patients (*P* < 0.05), with a 195% higher risk in those with hypertension versus without (bootstrap-adjusted OR = 2.945, 95% CI: 1.42–6.11; *P* = 0.004) (Table 4).

**TABLE 4:** Association between hypertension and risk of mortality in patients with COVID-19.

Variable		Analysis type	OR/aOR/bOR, 95%CI	*P*-value
Hypertensionl	No	Univariate analysis	1.0(reference)	–
	Yes		2.587(1.304, 5.131)	0.007
	No	Multivariate analysis (Model 1)^a^	1.0(reference)	–
	Yes		2.951(1.425, 6.114)	0.004
	No	Multivariate analysis (Model 2)^b^	1.0(reference)	–
	Yes		2.945(1.420, 6.107)	0.004
Smoking	No	Multivariate analysis (Model 1)^a^	1.0(reference)	–
	Yes		2.157(1.041, 4.469)	0.039
	No	Multivariate analysis (Model 2)^b^	1.0(reference)	–
	Yes		2.164(1.042, 4.497)	0.039
Alcohol	No	Multivariate analysis (Model 1)^a^	1.0(reference)	–
	Yes		4.257(0.709, 25.567)	0.113
	No	Multivariate analysis (Model 2)^b^	1.0(reference)	–
	Yes		4.225(0.701, 25.472)	0.116
BMI	Normal	Multivariate analysis (Model 1)^a^	1.0(reference)	–
	Overweight/Obese		0.800(0.379, 1.691)	0.559
	Normal	Multivariate analysis (Model 2)^b^	1.0(reference)	–
	Overweight/Obese		0.795(0.373, 1.693)	0.552
Diabetes	No	Multivariate analysis (Model 2)^b^	1.0(reference)	–
	Yes	1.053(0.454, 2.441)	0.905

(Model 1)^a^ refers to multivariate logistic regression analysis adjusted for “hypertension, age, and sex”.

(Model 2)^b^ refers to multivariate logistic regression analysis adjusted for “hypertension, age, sex, smoking, and alcohol consumption”.

### 3.3 Mediation analysis of the association between hypertension and risks of respiratory failure and mortality in COVID-19 patients

Mediation analysis of 47 lab parameters (e.g., CBC, metabolic panel, coagulation, D-dimer, troponin, BNP) revealed eosinophil count and eGFR as key mediators linking hypertension to respiratory failure and mortality in COVID-19 patients.

#### 3.3.1 Mediation analysis of the association between hypertension and risk of respiratory failure in COVID-19 patients

**FIGURE 4:**
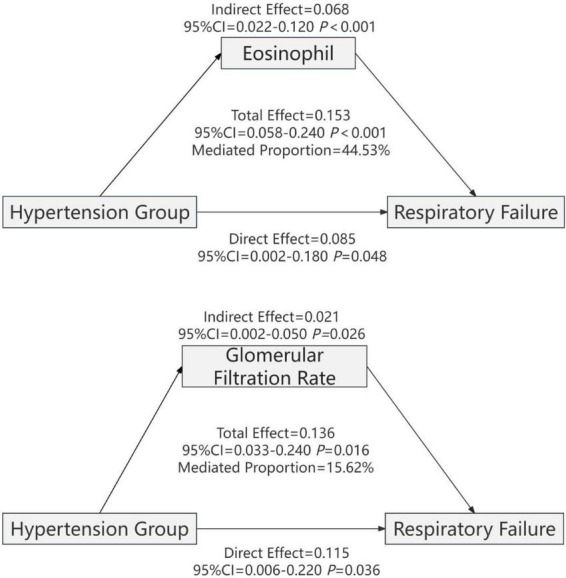
Mediating effect analysis of eosinophils and glomerular filtration rate in the association between hypertension comorbidity and risk of respiratory failure in COVID-19 patients. Note: Adjusted for BMI, smoking, alcohol consumption, and diabetes history.

Mediation analysis of 47 laboratory parameters revealed that eosinophil count and estimated glomerular filtration rate act as partial mediators in the association between hypertension and respiratory failure in patients with COVID-19 (Figure 4). Eosinophil count accounted for 44.53% of the total effect, with an indirect effect of β = 0.068 (95% CI: 0.022–0.120; *P* < 0.001) and a direct effect of β = 0.085 (95% CI: 0.002–0.180; *P* = 0.048). Estimated glomerular filtration rate (eGFR) accounted for 15.62% of the total effect, with an indirect effect of β = 0.021 (95% CI: 0.002–0.050; *P* = 0.026) and a direct effect of β = 0.115 (95% CI: 0.006–0.220; *P* = 0.036) (Table 5).

**TABLE 5:** Mediation analysis of the association between COVID-19 with hypertension and respiratory failure.

Variable	Indirect Effect	95%CI	*P*	Direct Effect	95%CI	*P*	Mediation effect value (%)
WBC	–0.024	–0.055, 0.01	0.118	0.162	0.048, 0.27	0.010	–17.81
NEUT	–0.024	–0.059, 0.01	0.158	0.173	0.073, 0.27	0.002	–16.23
lymphocytes	0.011	–0.004, 0.04	0.190	0.119	0.018, 0.22	0.016	8.146
NLR	–0.015	–0.061, 0.03	0.586	0.134	0.042, 0.23	0.004	–12.35
MO	–0.003	–0.016, 0.01	0.690	0.132	0.031, 0.22	0.020	–2.182
EOs	0.068	0.022, 0.12	< 2e-16	0.085	0.002, 0.18	0.048	44.53
BA	–0.006	-0.029, 0.03	0.606	0.136	0.042, 0.23	0.012	–32.44
RBC	0.001	–0.006, 0.01	0.800	0.128	0.024, 0.23	0.016	0.486
Hb	–0.002	–0.014, 0.01	0.798	0.131	0.031, 0.23	0.008	–1.295
MCV	0.003	–0.003, 0.04	0.270	0.127	0.025, 0.23	0.010	2.040
MCH	-0.004	–0.021, 0.02	0.644	0.132	0.040, 0.24	0.006	2.970
MCHC	-0.002	–0.047, 0.01	0.604	0.13	0.034, 0.25	0.006	–2.970
Hct	–0.001	–0.013, 0.01	0.882	0.129	0.032, 0.23	0.010	–0.584
PLT	4.37e-04	–0.006, 0.01	0.864	0.128	0.026, 0.23	0.010	0.339
CRP	0.008	–0.020, 0.04	0.628	0.136	0.025, 0.23	0.010	5.519
PCT	0.033	–0.100, 0.09	0.744	0.038	0.002, 0.20	0.034	46.746
ALT	0.014	(–7.73e04), 0.04	0.076	0.104	(–6.09e-04), 0.20	0.054	11.823
AST	0.001	–0.021, 0.02	0.876	0.126	0.022, 0.22	0.018	0.007
ALB	0.018	–0.015, 0.05	0.296	0.104	0.014, 0.20	0.018	14.760
GLB	0.006	–0.005, 0.02	0.330	0.116	0.017, 0.22	0.024	5.124
TBIL	5.62e-05	–0.008, 0.01	0.964	0.123	0.0141, 0.22	0.022	0.046
DBIL	0.013	–0.002, 0.04	0.104	0.109	0.009, 0.22	0.034	10.566
IBIL	0.001	–0.009, 0.01	0.852	0.122	0.027, 0.22	0.012	0.961
LDH	0.014	–0.011, 0.05	0.300	0.123	0.009, 0.23	0.036	9.984
CK	–0.004	–0.020, 0.04	0.774	0.145	0.040, 0.26	0.008	-2.552
CK-MB	0.004	–0.010, 0.03	0.632	0.142	0.020, 0.25	0.014	2.558
CREA	0.004	–0.006, 0.02	0.356	0.125	0.023, 0.23	0.364	2.961
eGFR	0.021	0.002, 0.05	0.026	0.115	0.006, 0.22	0.036	15.615
BUN	0.008	–0.015, 0.04	0.514	0.126	0.026, 0.23	0.014	0.061
GLU	–3.23E-04	–0.012, 0.001	0.640	0.130	0.034, 0.23	0.010	-0.250
TG	0.004	–0.010, 0.03	0.632	0.142	0.020, 0.25	0.014	2.558
CHO	–0.008	– 0.038, 0.02	0.514	0.176	0.045, 0.31	0.006	–4.582
K^+^	–0.0002	–0.010, 0.01	0.880	0.130	0.027, 0.24	0.020	–0.130
Na^+^	–0.003	–0.016, 0.02	0.704	0.132	0.027, 0.23	0.014	–2.073
Cl^–^	–0.004	–0.022, 0.01	0.544	0.133	0.035, 0.23	0.014	–3.368
Ca^2+^	0.008	-0.017, 0.03	0.486	0.129	0.032, 0.23	0.008	5.879
P	–0.001	–0.016, 0.01	0.598	0.152	0.036, 0.28	0.012	–0.813
AG	–0.001	–0.013, 0.01	0.904	0.129	0.029, 0.23	0.016	–0.397
OSMO	–0.012	-0.045, 0.01	0.280	0.130	0.026, 0.23	0.020	–10.314
PT	–0.001	–0.007, 0.01	0.850	0.130	0.025, 0.23	0.018	–1.058
TT	0.007	–0.018, 0.02	0.498	0.122	0.011, 0.23	0.030	5.578
APTT	0.001	–0.022, 0.01	0.830	0.128	0.032, 0.24	0.010	0.407
INR	–0.002	–0.011, 0.02	0.742	0.132	0.026, 0.24	0.016	–1.266
FIB	–0.005	–0.023, 0.01	0.508	0.132	0.027, 0.24	0.008	3.761
D-dimer	0.020	–0.020, 0.07	0.338	0.122	0.026, 0.22	0.010	13.94
BNP	–0.006	–0.063, 0.01	0.650	0.126	0.009, 0.23	0.036	4.592
Troponin	–0.011	-0.034, 0.00	0.170	0.133	0.037, 0.24	0.006	-8.894

#### 3.3.2 Mediation analysis of the association between hypertension and mortality risk in COVID-19 patients

**FIGURE 5:**
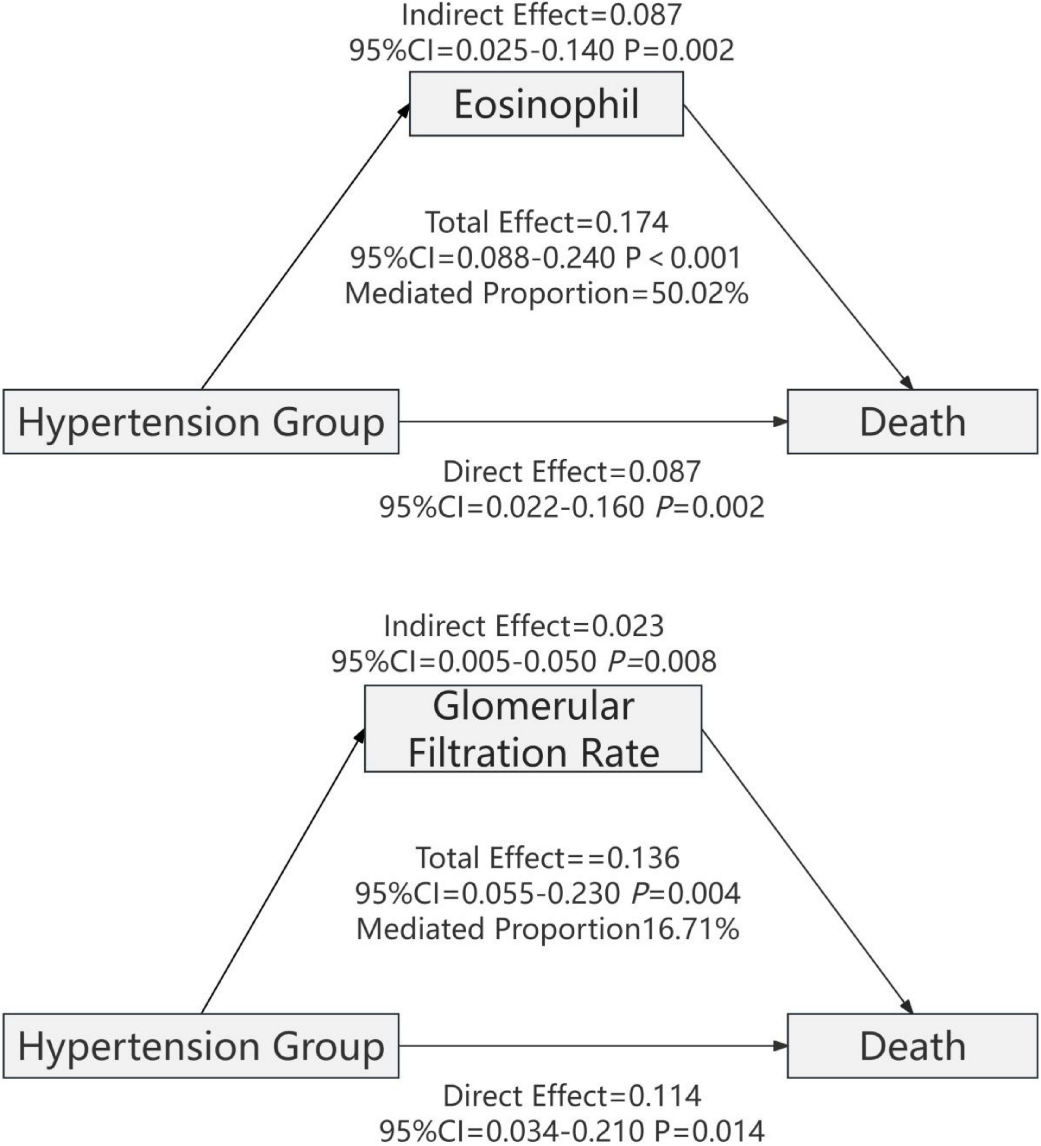
Mediation effect analysis of eosinophils and glomerular filtration rate respectively mediating the relationship between hypertension and mortality risk in patients with COVID-19. Variables such as BMI, smoking, drinking, and history of diabetes were adjusted.

Mediation analysis of 47 laboratory parameters revealed that eosinophil count and estimated glomerular filtration rate (eGFR) act as partial mediators in the association between hypertension and mortality risk in patients with COVID-19 (Figure 5). Eosinophil count accounted for 50.02% of the total effect, with an indirect effect of β = 0.087 (95% CI: 0.025–0.140; *P* = 0.002) and a direct effect of β = 0.087 (95% CI: 0.020–0.160; *P* = 0.002). Estimated glomerular filtration rate (eGFR) accounted for 16.71% of the total effect, with an indirect effect of β = 0.023 (95% CI: 0.005–0.050; *P* = 0.008) and a direct effect of β = 0.114 (95% CI: 0.034–0.210; *P* = 0.014) (Table 6).

**TABLE 6:** Mediation analysis of the association between COVID-19 with hypertension and mortality.

Variable	Indirect Effect	95%CI	*P*	Direct Effect	95%CI	*P*	Mediation effect value (%)
WBC	–0.019	–0.048, 0.00	0.130	0.161	0.067, 0.26	< 2e-16	–13.460
NEUT	–0.019	–0.049, 0.01	0.142	0.166	0.073, 0.26	0.002	–12.570
Lymphocytes	0.007	–0.005, 0.03	0.346	0.124	0.037, 0.21	0.006	5.158
NLR	–0.003	–0.011, 0.03	0.620	0.134	0.038, 0.22	0.006	–2.192
MO	–0.002	–0.013, 0.01	0.780	0.132	0.038, 0.21	0.008	–1.707
EOs	0.087	0.025, 0.14	0.002	0.087	0.022, 0.16	0.002	50.200
BA	–0.009	–0.028, 0.01	0.302	0.139	0.045, 0.23	0.002	–6.686
RBC	0.002	–0.005, 0.01	0.674	0.128	0.041, 0.22	0.004	1.391
Hb	–0.002	–0.014, 0.00	0.616	0.131	0.044, 0.22	0.004	–1.654
MCV	0.001	–0.003, 0.02	0.398	0.128	0.037, 0.21	0.002	0.388
MCH	0.005	–0.007, 0.03	0.462	0.125	0.037, 0.21	0.006	3.706
MCHC	–2.23e-05	–0.023, 0.00	0.530	0.129	0.039, 0.23	0.002	–0.017
Hct	3.60e-04	(-9.965e-3), 0.01	0.084	0.130	0.048, 0.22	0.004	–0.279
PLT	0.001	–0.005, 0.01	0.750	0.127	0.039, 0.22	< 2e-16	0.524
CRP	0.005	–0.016, 0.03	0.622	0.132	0.037, 0.22	0.006	3.859
PCT	1.84e-06	–0.082, 0.00	0.404	0.131	0.033, 0.26	0.010	0.001
ALT	0.004	–0.010, 0.02	0.552	0.112	0.029, 0.21	0.008	3.206
AST	4.08e-04	–0.009, 0.02	0.918	0.124	0.036, 0.22	0.010	0.327
ALB	0.011	–0.008, 0.04	0.228	0.110	0.021, 0.20	0.008	9.110
GLB	0.005	–0.006, 0.02	0.346	0.118	0.023, 0.21	0.012	4.162
TBIL	0.000	–0.010, 0.01	0.960	0.122	0.036, 0.21	< 2e-16	0.131
DBIL	0.009	–0.003, 0.03	0.178	0.113	0.016, 0.22	0.028	7.624
IBIL	0.003	–0.006, 0.01	0.648	0.121	0.025, 0.21	0.010	2.331
LDH	0.013	–0.012, 0.04	0.264	0.116	0.012, 0.23	0.026	9.917
CK	–0.015	–0.037, 0.00	0.060	0.144	0.036, 0.25	0.006	–11.480
CK-MB	0.007	–0.020, 0.05	0.680	0.122	0.034, 0.23	0.008	5.643
CREA	0.005	–0.004, 0.02	0.252	0.125	0.038, 0.22	0.008	3.485
eGFR	0.023	0.005, 0.05	0.008	0.114	0.034, 0.21	0.014	16.708
BUN	0.008	–0.044	0.554	0.127	0.033, 0.22	0.006	5.636
GLU	1.05e-04	(–9.33e-03), 0.01	0.842	0.130	0.035, 0.23	0.004	0.081
TG	0.007	–0.012, 0.03	0.422	0.133	0.012, 0.26	0.026	5.275
CHO	–2.62e-05	-0.014, 0.01	0.940	0.139	0.009, 0.27	0.030	–0.019
K^+^	4.64e-04	–0.009, 0.01	0.870	0.129	0.044, 0.22	< 2e-16	0.358
Na^+^	0.009	–0.004, 0.04	0.298	0.130	0.030, 0.21	0.006	6.658
Cl^–^	–0.005	–0.025, 0.01	0.468	0.135	0.052, 0.23	0.004	–4.064
Ca^2+^	0.004	–0.011, 0.02	0.572	0.130	0.041, 0.23	0.014	3.336
P	–0.003	–0.021, 0.01	0.620	0.154	0.052, 0.26	0.002	–1.758
AG	–0.001	–0.018, 0.01	0.860	0.132	0.052, 0.23	0.002	–0.777
OSMO	–0.009	-0.040, 0.01	0.342	0.133	0.041, 0.22	0.002	–7.208
PT	–9.97e-07	–0.006, 0.01	0.974	0.120	0.026, 0.21	0.014	–8.31e-04
TT	0.017	(–3.82e-04), 0.04	0.056	0.105	0.016, 0.20	0.026	13.993
APTT	–0.001	–0.003, 0.05	0.580	0.121	0.030, 0.22	0.006	–0.619
INR	–0.001	–0.008, 0.01	0.880	0.120	0.030, 0.21	0.002	–0.627
FIB	0.001	–0.006, 0.01	0.822	0.118	0.026, 0.21	0.012	0.008
D-dimer	0.022	–0.022, 0.07	0.378	0.132	0.035, 0.22	0.002	14.290
BNP	–0.003	–0.042, 0.02	0.616	0.124	0.038, 0.24	0.014	–2.802
Troponin	–0.015	–0.048, 0.00	0.118	0.138	0.042, 0.24	0.006	–11.950

### 3.4 Subgroup analysis

To examine whether the association of hypertension with the risks of respiratory failure and mortality in patients with COVID-19 varied across subgroups defined by key demographic and clinical characteristics, we performed subgroup analyses by including interaction terms between hypertension and each subgroup variable in the multivariable logistic regression models. The subgroup variables included age (< 65 vs. ≥ 65 years), sex (female vs. male), smoking history (no vs. yes), alcohol consumption (no vs. yes), and BMI (normal vs. overweight/obese).

#### 3.4.1 Subgroup analysis of the association between hypertension and respiratory failure in COVID-19 patients

**FIGURE 6:**
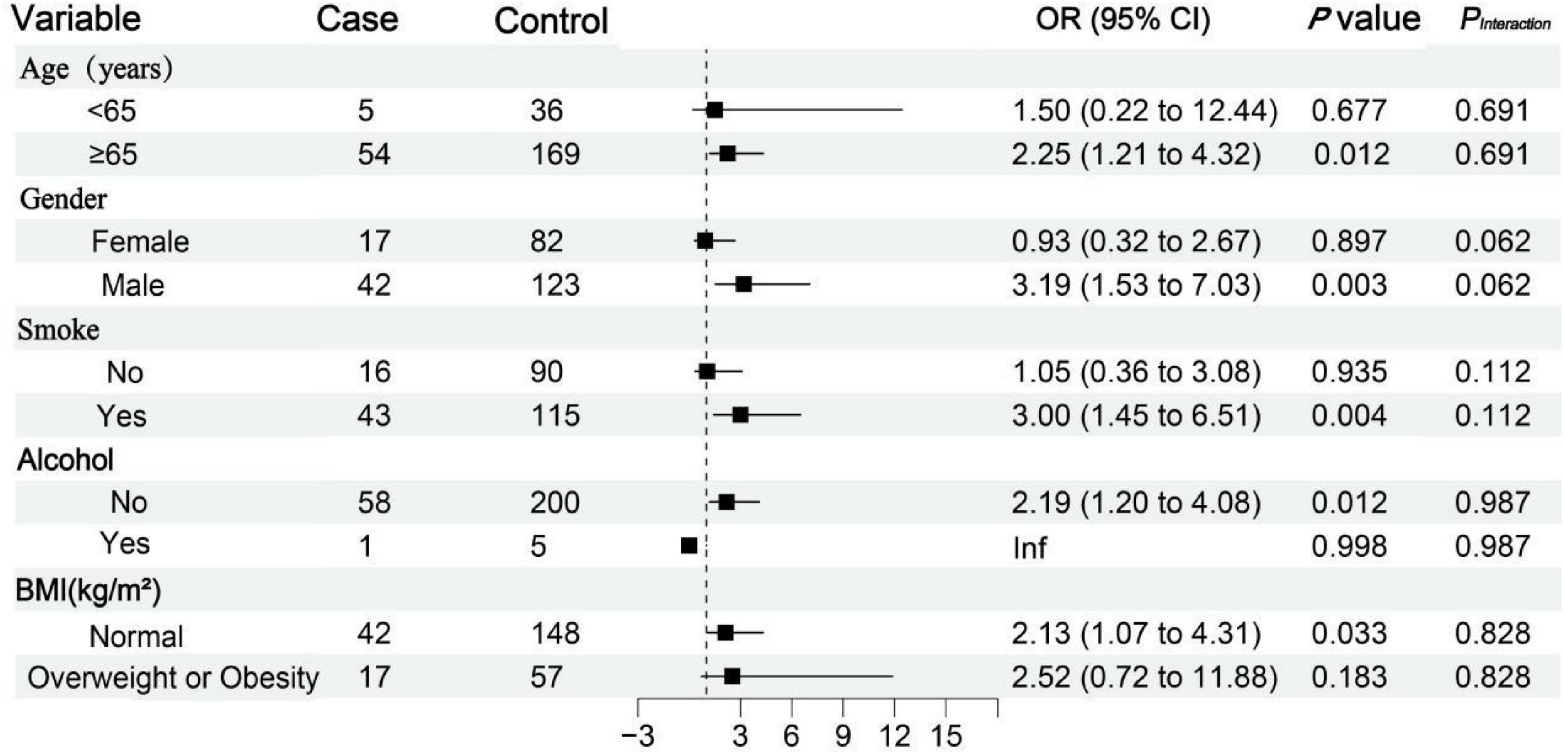
Subgroup analysis of the association between hypertension and respiratory failure in patients with COVID-19. BMI: body mass index.

Subgroup analysis revealed that the association between hypertension and respiratory failure in patients with COVID-19 was appeared stronger in subgroups of patients aged ≥ 65 years (OR = 2.25, 95% CI: 1.21–4.32; *P* = 0.012), males (OR = 3.19, 95% CI: 1.53–7.03; *P* = 0.003), smokers (OR = 3.00, 95% CI: 1.45–6.51; *P* = 0.004), alcohol consumers (OR = 2.19, 95% CI: 1.20–4.08; *P* = 0.012), and those with normal BMI (OR = 2.13, 95% CI: 1.07–4.31; *P* = 0.033), suggesting heterogeneity in the effect of hypertension across patient subgroups. However, formal tests for interaction were not statistically significant (Figure 6).

#### 3.4.2 Subgroup analysis of the association between hypertension and mortality risk in COVID-19 patients

**FIGURE 7:**
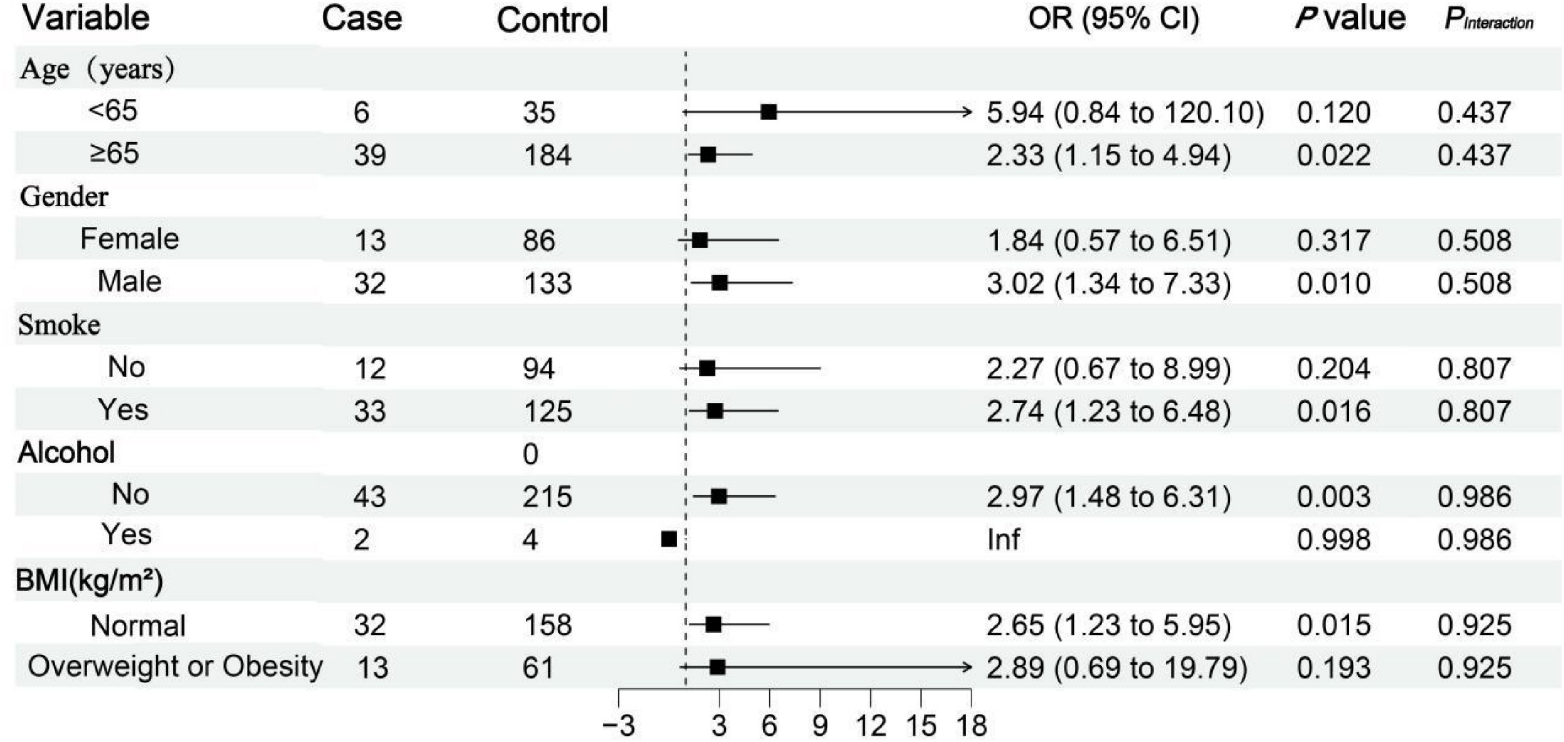
Subgroup analysis of the association between hypertension and mortality in patients with COVID-19. BMI, body mass index.

Subgroup analysis showed that the association between hypertension and mortality risk in patients with COVID-19 appeared stronger in those aged ≥ 65 years (OR = 2.33, 95% CI: 1.51–4.94; *P* = 0.022), males (OR = 3.02, 95% CI: 1.34–7.33; *P* = 0.010), smokers (OR = 2.74, 95% CI: 1.23–6.48; *P* = 0.016), alcohol consumers (OR = 2.97, 95% CI: 1.48–6.31; *P* = 0.003), and individuals with normal BMI (OR = 2.65, 95% CI: 1.23–5.95; *P* = 0.015), suggesting potential heterogeneity in the effect of hypertension across subgroups. However, formal tests for interaction were not statistically significant (Figure 7).

### 3.5 Development of a predictive model for respiratory failure and mortality in COVID-19 patients with hypertension

#### 3.5.1 Development of a predictive model for respiratory failure in COVID-19 patients with hypertension

**FIGURE 8:**
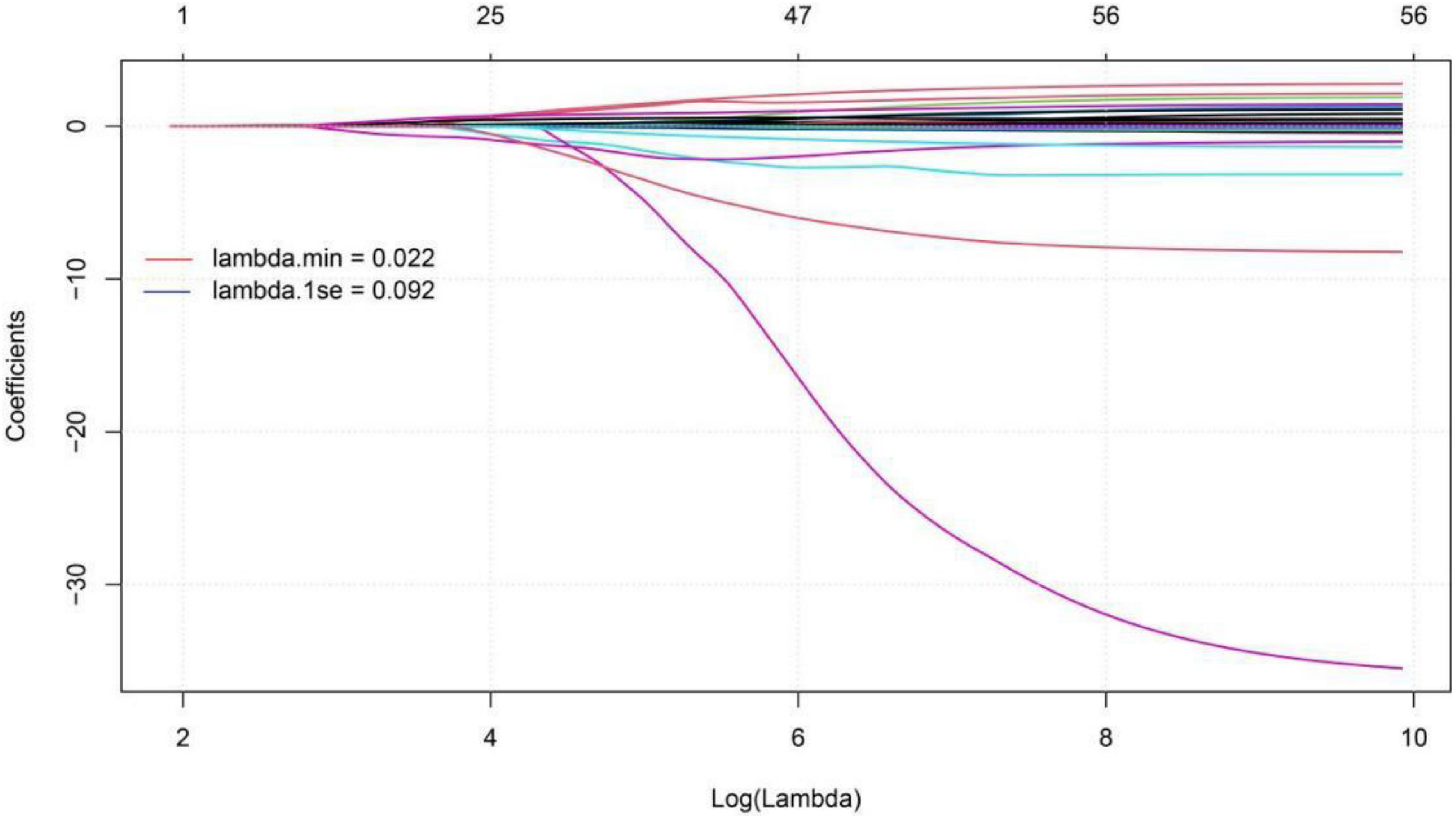
Lasso coefficient path diagram of respiratory failure in COVID-19 patients with hypertension.

**FIGURE 9:**
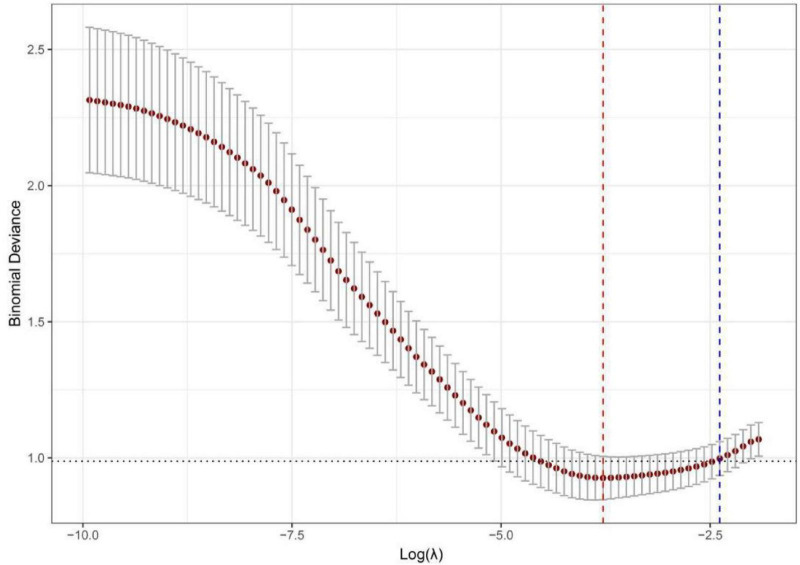
Lasso cross-validation error curve diagram of respiratory failure in COVID-19 patients with hypertension.

**FIGURE 10:**
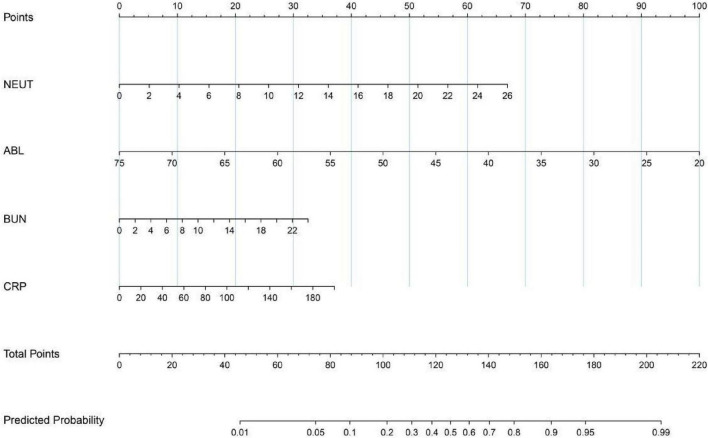
Nomogram for respiratory failure in COVID-19 patients with hypertension.

To predict the risk of respiratory failure in COVID-19 patients with hypertension, we employed LASSO regression to select predictors from 55 candidate variables, including demographic, clinical, and laboratory parameters.The optimal regularization parameter λ was determined via 10-fold cross-validation, yielding λmin = 0.023 and λ1se = 0.092. The model corresponding to λ1se—which maintains cross-validated binomial deviance within one standard error of the minimum (0.998 ± 0.062)—retained only 4 predictors with non-zero coefficients, offering a more parsimonious and strongly regularized model with enhanced generalizability compared to the λmin model (21 predictors). Therefore, the λ1se model was selected to define the final set of predictors (Figures 8, 9). The four features selected for inclusion in the subsequent predictive model—and their corresponding coefficients in the LASSO model—were: neutrophil count (β = 0.043), C-reactive protein (CRP) (β = 0.006), albumin (β = -0.006), and blood urea nitrogen (BUN) (β < 0.001). Based on the key predictors selected by LASSO regression (neutrophil count, CRP, albumin, and BUN), We constructed a nomogram to estimate the likelihood of respiratory failure among COVID-19 patients suffering from hypertension. The horizontal axis of the nomogram corresponds to the value ranges of the four variables—neutrophil count, albumin, BUN, and CRP—as shown in Figure 10. For each variable, its specific value is projected vertically upward to the “Points” axis to assign a score; the four scores are summed as “Total Points” and then mapped to the “Predicted Probability” axis to estimate the risk of respiratory failure—allowing rapid, intuitive risk assessment in clinical practice.

#### 3.5.2 Development of a predictive model for mortality in COVID-19 patients with hypertension

**FIGURE 11:**
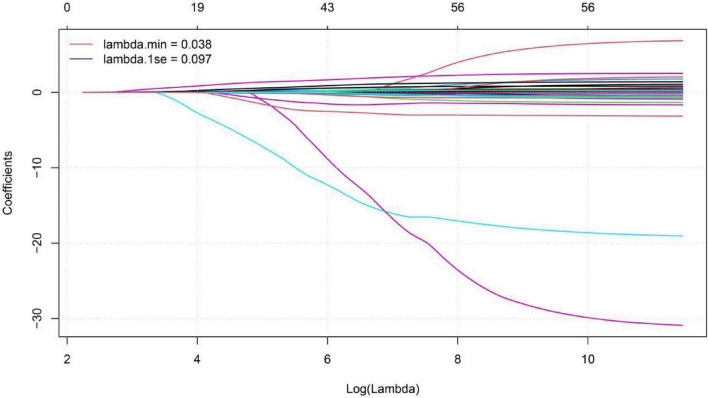
Lasso coefficient path diagram of Mortality risk in COVID-19 patients with hypertension.

**FIGURE 12:**
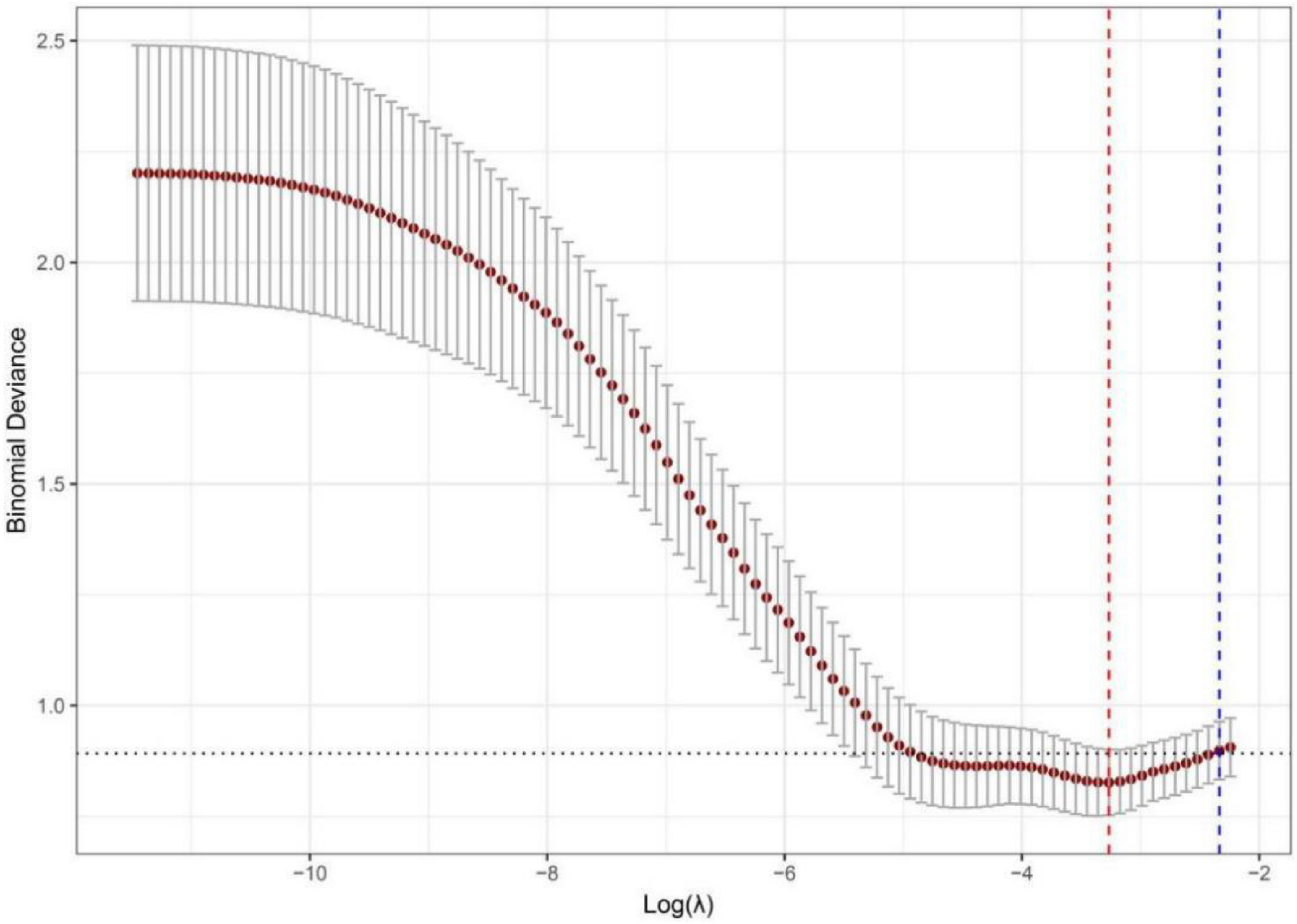
Lasso cross-validation error curve diagram of mortality risk in COVID-19 patients with hypertension.

**FIGURE 13:**
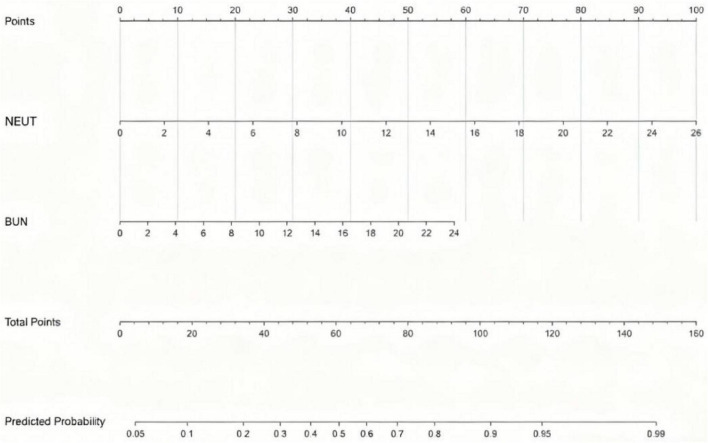
Nomogram for mortality risk in COVID-19 patients with hypertension.

To predict the risk of mortality in patients with COVID-19 and hypertension, we employed LASSO regression to select predictors from 55 candidate variables, including demographic, clinical, and laboratory measures. The optimal regularization parameter λ was determined via 10-fold cross-validation, yielding λ.min = 0.038 and λ0.1se = 0.097. The λ0.1se model—whose cross-validated binomial deviance (0.898 ± 0.065) lies within one standard error of the minimum—retained only 2 predictors with non-zero coefficients, yielding a more parsimonious and strongly regularized model with improved generalizability compared to the λmin model (8 predictors). Therefore, the λ0.1se model was selected to define the final predictor set (Figures 11, 12). The final model included two LASSO-selected predictors: BUN (β = 0.018) and neutrophil count (β = 0.002). A nomogram was then developed to predict mortality risk in patients with COVID-19 and hypertension. The horizontal axis of the nomogram corresponds to the value ranges of the two predictors—neutrophil count and BUN—with each value projected upward to the “Points” axis to assign a score (Figure 13). The “Total Points” were calculated by adding up the points assigned to each of the four predictors, and then aligned with the bottom “Predicted Probability” axis to determine the individual’s probability of death—facilitating rapid and intuitive risk assessment in clinical practice.

### 3.6 ROC curve analysis

**FIGURE 14:**
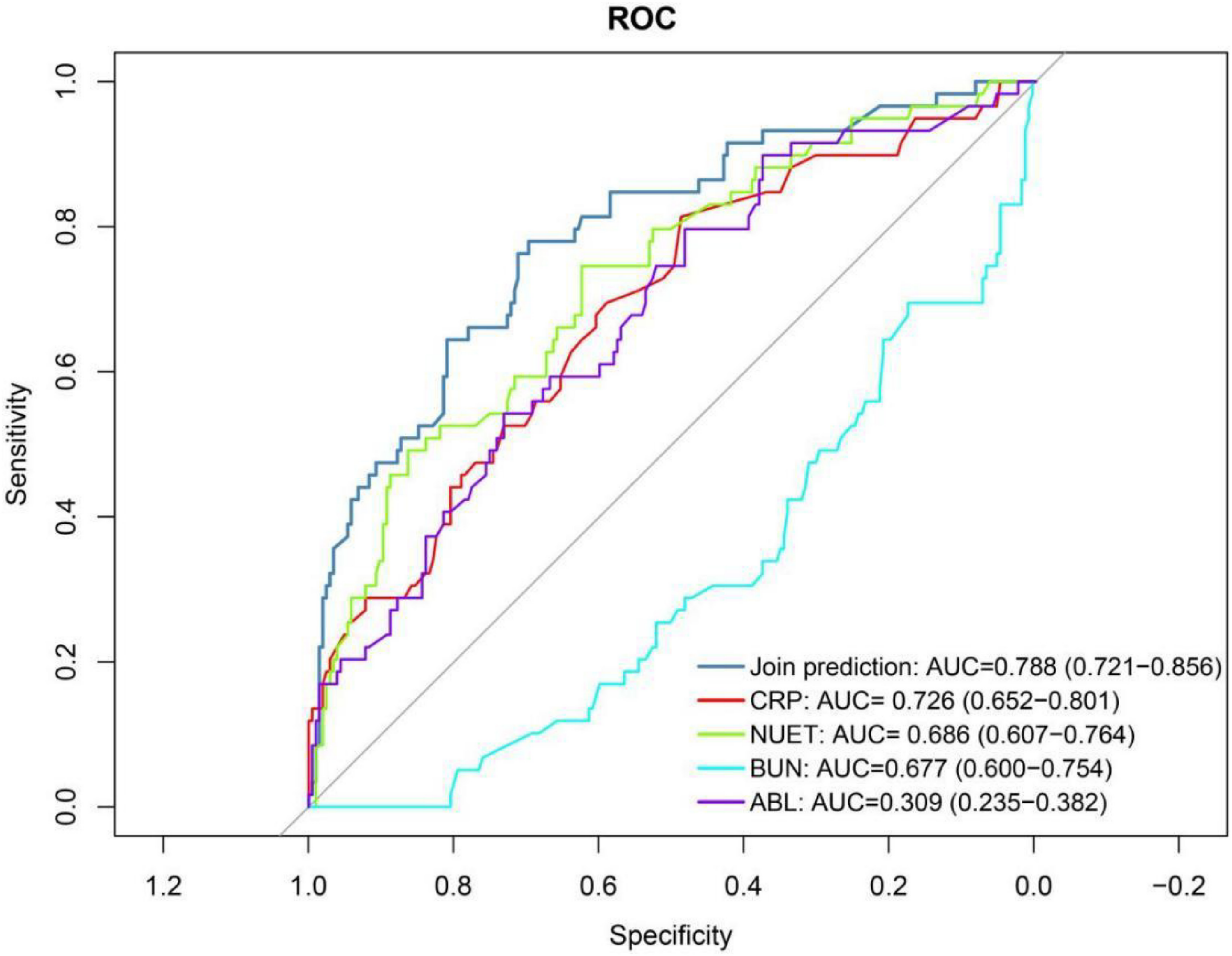
ROC curve analysis of respiratory failure in COVID-19 patients with hypertension. NEUT, Neutrophil; BUN, Blood Urea Nitrogen; ABL, Albumin.

**FIGURE 15:**
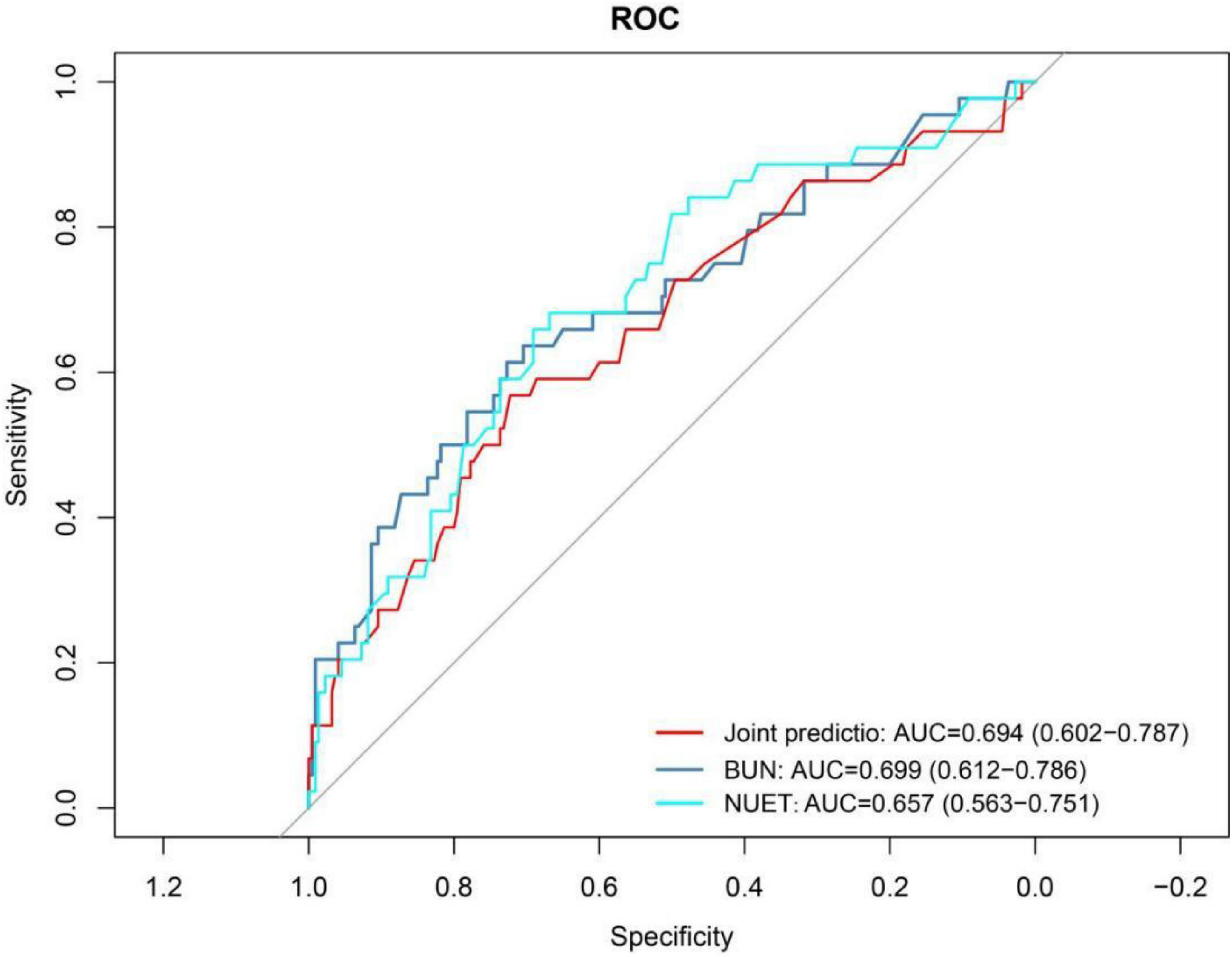
ROC curve analysis of mortality risk in COVID-19 patients with hypertension. NEUT, Neutrophil; BUN, Blood Urea Nitrogen.

Variables selected by LASSO regression were used to build a multivariable logistic regression model. Its predictive performance was assessed via the ROC curve and AUC, with *P* < 0.05 indicating statistical significance. Results showed that the four-predictor model (neutrophil count, CRP, albumin, and BUN) achieved an AUC of 0.788 (95% CI: 0.721–0.856), indicating good predictive performance for respiratory failure (Figure 14). In contrast, the two-predictor model (neutrophil count and BUN) yielded an AUC of 0.694 (95% CI: 0.602–0.787), reflecting modest predictive ability for mortality risk (Figure 15).

## 4 Discussion

In this single-center retrospective cohort study, we investigated the prognostic implications of pre-existing hypertension in hospitalized patients with COVID-19. The data collection for this study spanned from October 2022 to May 2023, a period corresponding to China’s transition to an endemic management phase dominated by Omicron variants. Although the viral pathogenicity during this phase differed from that observed in earlier waves of infection, hypertension remains a stable risk factor contributing to immune-metabolic dysregulation and decreased physiological reserve. Consequently, the findings of this study retain considerable relevance for real-world risk stratification in contemporary clinical practice. After rigorous propensity score matching to balance for key confounders (e.g., age, sex) and comprehensive multivariable adjustment, we confirm that hypertension is an independent risk factor for both respiratory failure and in-hospital mortality in patients with COVID-19. Specifically, patients with hypertension exhibited an approximately 120% higher risk of respiratory failure (adjusted OR = 2.196, 95% CI: 1.170–4.120) and a nearly 195% increased risk of in-hospital death (adjusted OR = 2.945, 95% CI: 1.420–6.107). This association remained robust after adjusting for smoking, alcohol use, BMI, and diabetes—addressing a common limitation of prior studies with inadequate confounder control (17, 18), and reinforcing hypertension as an independent prognostic factor for improved clinical risk stratification. Hypertension is a common comorbidity among patients with COVID-19, and those with hypertension exhibit significantly higher rates of severe disease and death compared to those without hypertension (4, 10–16). Evidence suggests that hypertension is not associated with increased susceptibility to SARS-CoV-2 infection; however, older age and irregular antihypertensive treatment are independent predictors of in-hospital mortality in COVID-19 patients with hypertension—and substantially contribute to increased healthcare costs (13, 23, 24). Under physiological conditions, angiotensin-converting enzyme 2 (ACE2) converts angiotensin II (Ang II) into angiotensin-(1–7) [Ang-(1–7)], a peptide with vasodilatory, antiproliferative, and antioxidant properties, thereby maintaining cardiovascular homeostasis (25, 26). The cellular entry of SARS-CoV-2 is mediated by its binding to the ACE2 receptor, subsequently reducing ACE2 levels and increasing angiotensin II (Ang II) levels, thereby disrupting the physiological balance of the renin–angiotensin–aldosterone system (RAAS). This leads to excessive activation of the Ang II type 1 receptor (AT1R) in pulmonary cells, ultimately triggering and exacerbating lung injury (25–28). Meanwhile, reduced ACE2 levels lead to diminished generation of Ang-(1–7), thereby weakening the counter-regulatory effects of the Ang-(1–7)/Mas axis against the deleterious actions of Ang II—such as inflammation, fibrosis, and hypertrophy—resulting in a vicious cycle: “ACE2 downregulation → Ang-(1–7) reduction → predominant Ang II activation → exacerbated myocardial remodeling and dysfunction,” which further aggravates structural and functional damage to the cardiovascular system (29, 30). Therefore, the ACE2-mediated imbalance in Ang-(1–7)/Ang II metabolism may serve as a critical pathological link between hypertension and adverse outcomes in COVID-19, and also provides a theoretical basis for RAAS-targeted interventions (25).

This study, based on mediation analysis using the Bootstrap method (with 1,000 resampling iterations), indicates that both eosinophil count (Eos) and eGFR exert partial mediating effects in this association. The data indicate that eosinophil count (Eos) mediated 44.5 and 50.0% of the total effect of hypertension on respiratory failure and mortality, respectively, whereas eGFR accounted for 15.6 and 16.7%. These findings provide novel mechanistic insights into the pathways linking hypertension to adverse outcomes in patients with COVID-19. Eosinopenia is frequently observed in COVID-19 and has been consistently associated with greater disease severity and a worse prognosis in multiple clinical studies (15, 17). A systematic review encompassing 4,663 patients reported that 58.4% of individuals exhibited eosinopenia (31). Furthermore, a retrospective cohort study demonstrated that peripheral blood eosinophil counts significantly decreased with increasing disease severity in COVID-19 (32). Lower eosinophil levels at admission are often associated with more extensive pulmonary involvement and prolonged hospitalization; conversely, a gradual recovery of eosinophil counts during treatment frequently parallels clinical improvement, underscoring the diagnostic, prognostic, and dynamic monitoring value of this parameter (33). The specific mechanisms by which eosinophils participate in antiviral immunity remain incompletely understood. Studies suggest that eosinophils may contribute to host defense through the following pathways: inhibiting viral replication via the release of cationic proteins and reactive oxygen species; modulating adaptive immunity as antigen-presenting cells; and enhancing viral clearance through Toll-like receptor (TLR)-mediated recruitment to the lungs in respiratory viral infection models (34–36). Additionally, research has shown that the type 2 immune cytokine IL-13 can downregulate ACE2 and TMPRSS2 expression in airway epithelial cells, thereby inhibiting viral entry. As effector cells in type 2 immunity, eosinophils may play a synergistic protective role in this context (37). However, these mechanisms have not been directly validated in the population data of the present study. Due to the limitations of the study design and the biomarkers measured, the current results only reflect statistical associations between eosinophil levels and clinical phenotypes/outcomes. They do not confirm a direct antiviral effect mediated by specific molecular pathways (e.g., TLR7–MyD88) or via modulation of the ACE2/RAAS axis. Future research should build on the foundational clues provided by this study and systematically validate these hypotheses through prospective cohort studies, integrated multi-omics analyses, and targeted mechanistic experiments.

Impaired renal function upon admission (often defined as eGFR < 60 mL/min/1.73 m^2^) is a significant predictor of poor outcomes in COVID-19 patients (22, 38). Extensive studies have shown that such patients are more likely to require high-flow oxygen therapy, endotracheal intubation, or even extracorporeal membrane oxygenation (ECMO), and have a substantially increased risk of in-hospital mortality (21, 39, 40). In COVID-19 patients with hypertension, renal impairment is more common and often coexists with diabetes and cardiovascular disease, creating a multimorbidity profile that significantly worsens disease severity and outcomes (40). Studies have shown that hypertension can further impair nitric oxide bioavailability and renal water-sodium regulation through various cellular and inflammatory mediators, thereby promoting renal fibrosis and functional decline (41). Mechanistically, diabetes impairs immunity and promotes hyperglycemia, increasing susceptibility to infection and risk of organ damage (42). Therefore, the co-occurrence of hypertension, diabetes, and renal impairment generally signals a heavier disease burden and worse prognosis, warranting closer monitoring and personalized management in this high-risk group. Notably, even among patients without pre-existing chronic kidney disease (CKD), a low eGFR at admission independently predicts progression to severe disease and all-cause mortality (43, 44). This suggests that eGFR reflects not only renal dysfunction but also broader systemic impairment, such as endothelial injury, chronic inflammation, and immune dysregulation (45, 46). In SARS-CoV-2 infection, renal insufficiency is frequently accompanied by hypercoagulability, metabolic abnormalities, and immune dysregulation, and these factors collectively increase the risk of respiratory failure and death (47, 48). In hypertensive patients, endothelial dysfunction and overactivation of the renin–angiotensin system reinforce each other, creating a vicious cycle and contributing to worse prognosis (45, 49). Large-scale studies have shown that even in individuals with normal urinary albumin excretion (UACR < 10 mg/g), those with an eGFR of 45–59 mL/min/1.73 m^2^ still have a 30% increased risk of hospitalization (43), indicating that mild renal impairment serves as an early warning sign of poor prognosis. Therefore, routine assessment of eGFR upon admission helps to quickly identify high-risk patients, enabling timely intensified monitoring and targeted interventions to improve clinical outcomes. Multiple studies have demonstrated that inflammatory and nutrition-related biomarkers hold significant value in predicting progression to severe disease or death in patients with COVID-19 (50, 51). Neutrophils, as key effector cells of innate immunity, exhibit aberrant activation closely associated with disease severity. They can exacerbate systemic inflammatory responses and promote tissue damage by releasing inflammatory mediators (52). Among COVID-19 patients, those with neutrophilia demonstrate a higher incidence of severe illness, indicating a close correlation with disease progression (53). C-reactive protein (CRP), as a typical acute-phase reactant, shows a significant correlation between elevated levels and adverse clinical outcomes in patients with COVID-19 (51). Albumin, a negative acute-phase protein, not only reflects the deterioration of nutritional status but also serves as an indicator predicting severe progression of COVID-19, demonstrating high sensitivity in determining mortality or predicting ICU admission rates (54). Studies confirm that dynamic monitoring of serum albumin levels in patients with COVID-19 pneumonia can be used as a practical biomarker to assess the progression of the “cytokine storm” and predict mortality risk, highlighting its valuable role in clinical surveillance (55). Moreover, the CRP to albumin ratio (CAR), which integrates both inflammatory activation and nutritional depletion, demonstrates significant clinical utility in distinguishing mild from severe cases and predicting mortality risk (51, 56). Current evidence indicates that, in COVID-19 patients with hypertension, BUN, albumin, and their derived ratio—the BUN-to-albumin ratio (BAR) are all significantly associated with in-hospital mortality risk (57, 58). Notably, BAR integrates information on renal compensatory status and nutritional/inflammatory burden, demonstrating superior prognostic discrimination compared to its individual components (58). Multiple studies have confirmed that the BAR is an independent risk factor for predicting in-hospital mortality. For example, a retrospective study including 1,027 patients found that for every 1-unit increase in BAR, the mortality risk increased by 6% (adjusted OR = 1.06, 95% CI: 1.03–1.08, *P* < 0.001) (59), indicating the practical value of this simple indicator in clinical risk stratification. In summary, the composite indicator model comprising neutrophils, CRP, albumin, and BUN integrates inflammatory, nutritional, and renal function status, providing solid evidence-based support for risk stratification of respiratory failure and early intervention in COVID-19 patients with hypertension.

The findings of this study support considering hypertension as a significant risk stratification factor in COVID-19 patients. As demonstrated in a recent multicenter randomized controlled trial, proactive adjunctive interventions can effectively improve respiratory function in patients with severe COVID-19 (60). This underscores the broader principle that early identification and timely intervention are paramount for optimizing patient outcomes. Based on the mediation analysis, close monitoring of dynamic changes in eosinophil counts, early identification of immune dysregulation, and implementing interventions to protect renal function may contribute to improve clinical outcomes of hypertensive patients with COVID-19. Meanwhile, a joint predictive model integrating four routinely measured biomarkers—neutrophil count, CRP, serum albumin, and BUN—provides a practical tool for the early clinical identification of high-risk patients. Given their ease and rapid turnaround in standard laboratory testing, dynamic monitoring of these biomarkers enables effective prediction of respiratory failure risk, facilitates timely therapeutic adjustments, and may ultimately contribute to improved clinical outcomes.

Several limitations of the present study warrant consideration. (1) Caution is warranted when extrapolating these results to broader populations, given the single-center retrospective design. (2) The relatively small sample size may affect the statistical power of the tests. (3) The lack of detailed information on patients’ blood pressure control status and specific antihypertensive medications used makes it difficult to further explore the impact of treatment on prognosis. (4) The precise disease stage at hospital admission could not be accurately determined for all patients, potentially introducing information bias and attenuating the observed associations between laboratory markers and clinical outcomes. (5) Although propensity score matching and multivariate adjustments have controlled for known confounding factors as much as possible, unmeasured factors such as vaccination status and SARS-CoV-2 viral subtypes may still introduce residual confounding. Future studies will need to incorporate detailed pharmacological and virological data to further disentangle independent effects.

## 5 Future perspectives

To validate and generalize our findings, Future studies should establish multicenter prospective cohorts across diverse viral evolutionary stages and healthcare environments, integrating ambulatory blood pressure monitoring, detailed medication records, and longitudinal biomarker measurements at multiple time points. In-depth investigations are needed to elucidate the causal pathways between eosinophil functional states (e.g., activation or degranulation markers) and eGFR decline. Advanced techniques such as single-cell sequencing and proteomics should be employed to decipher the interaction mechanisms of the “hypertension–immune–kidney axis.” Additionally, a dynamic prediction model integrating clinical, laboratory, and imaging features should be developed to facilitate the implementation of personalized interventions.

## 6 Conclusion

By comparing the clinical characteristics and outcomes of COVID-19 patients with versus without hypertension, this study confirms that hypertension is significantly associated with increased risks of respiratory failure and in-hospital mortality. Furthermore, mediation analysis reveals that reduced eosinophil count and declining eGFR partially mediate serve as key mediators in the association between hypertension and adverse outcomes. A predictive model based on admission laboratory parameters demonstrated that the combination of four markers—absolute neutrophil count, CRP, serum albumin, and blood urea nitrogen (BUN)—exhibits moderate predictive performance for respiratory failure, which facilitates the prompt recognition of individuals susceptible to adverse outcomes in clinical settings. These findings hold significant clinical implications for risk stratification, individualized treatment decision-making, and prognosis improvement in patients with COVID-19 and comorbid hypertension. Given the limitations of this study, including its single-center design, retrospective nature, and limited post-matching sample size, the reported associations and predictive models require further validation in large-scale, multicenter cohort studies across diverse viral evolution stages and healthcare settings to confirm their external applicability. Furthermore, in-depth exploration of the specific mechanisms by which eosinophils and renal function contribute to the pathophysiology of COVID-19, combined with rigorous assessment of the efficacy of targeted interventions, holds promise for further improving clinical outcomes in hypertensive patients with COVID-19.
